# In This Issue

**Published:** 2008

## COMMENTARY: SYSTEMS BIOLOGY AND ITS RELEVANCE TO ALCOHOL RESEARCH

The effects of alcohol on the body are complex and can be studied on an environmental as well as intrinsic level. Alcohol-induced disorders, including organ damage and addiction, reflect the genetic and epigenetic makeup and the cumulative responses to alcohol exposure over time. At the molecular level, the effects of alcohol and its metabolites are the consequences of changes in DNA, RNA, proteins, metabolites, and other molecules. At the systems level, alcohol affects a variety of organs, biochemical or signaling pathways, and biological processes. This article by Drs. Q. Max Guo and Sam Zakhari is a commentary on the benefits of studying the effects of alcohol abuse using a systems biology approach. Alcohol research using a systems biology approach will prove fruitful in unraveling the mechanisms of complex alcohol-induced disorders. (pp. 5–10)

## NIH ROADMAP FOR MEDICAL RESEARCH

In an effort to transform the Nation’s medical research capabilities and improve the translation of research into practice, the National Institutes of Health has designed a Roadmap for Medical Research, consisting of three major themes—new pathways to discovery, research teams of the future, and reengineering the clinical research enterprise. This sidebar by Ms. Lori Wolfgang Kantor describes the initiatives within each theme. (pp. 12–13)

## SYSTEMS GENETICS OF ALCOHOLISM

Alcoholism results from a complex interaction of genetic as well as environmental and social factors. Increasing interest in the heritability of alcoholism has resulted in numerous studies of how single genes, as well as an individual’s entire genetic content, influence alcoholism risk. This article by Ms. Chantel D. Sloan, Ms. Vicki Sayarath, and Dr. Jason H. Moore examines how technological and statistical tools can be used to further the understanding of alcoholism’s genetic basis as well as key findings from candidate gene and genome-wide studies. These studies confirm the role of genetics in the development of alcoholism and elucidate the need for a systems-based approach to the study of the genetic basis of the disease. (pp. 14–25)

## METABOLOMICS IN ALCOHOL RESEARCH AND DRUG DEVELOPMENT

Metabolomics—a systems biology approach to characterizing metabolites produced in biochemical pathways—has contributed to many studies of disease progression and treatment. Metabolomic approaches are particularly useful because the metabolome—the entire set of all metabolites in a cell or tissue—represents the cell’s or tissue’s functional status more closely than, for example, the genome, because it responds to induced chemical or environmental changes and reflects the overall effects of all genetic and environmental alterations. The article by Drs. George G. Harrigan, Greg Maguire, and Laszlo Boros discusses how metabolomic approaches also may be applicable in alcohol research, using the examples of lipid metabolism and of metabolic pathways involving the vitamin thiamine, both of which are altered by excessive alcohol use. By further increasing the number and types of metabolites that can be measured in a given biological sample, metabolomic approaches may be able to help define the role of the many different metabolic pathways impacted by alcohol abuse and provide a means to support discovery and development of novel medications for the treatment of alcoholism and related conditions. (pp. 26–35)

## PROTEOMIC APPROACHES FOR STUDYING ALCOHOLISM AND ALCOHOL-INDUCED ORGAN DAMAGE

Proteomics research—the study of all the proteins in a cell, tissue, or organism—increasingly is being used in alcohol research to study alcohol’s effects on the body. This article by Dr. Susanne Hiller-Sturmhöfel, Mr. Josip Sobin, and Dr. R. Dayne Mayfield introduces numerous proteomic approaches, including shotgun strategies, assays to determine protein–protein interactions, and in silico analyses. All of these strategies are being used to identify the proteins that are affected by alcohol. Moreover, the results of such analyses may help in the identification of biomarkers of alcoholism and susceptibility to alcohol-induced tissue damage as well as of proteins that may eventually become therapeutic targets for alcoholism. (pp. 36–48)

## MATHEMATICAL MODELING OF COMPLEX BIOLOGICAL SYSTEMS: FROM PARTS LISTS TO UNDERSTANDING SYSTEMS BEHAVIOR

Recent technological advances that allow for processing of numerous samples simultaneously have enabled researchers to monitor complex cellular processes on a molecular level. This article by Hans Peter Fischer discusses mathematical and computational model approaches to systems biology. The article explores the “-omics” technologies-transcriptomics, proteomics, and metabolomics. The information gained from these experimental approaches should shed new light on the biochemical reactions that control functions such as metabolism, cell growth, reproduction, and the stress response. (pp. 49–59)

## SYSTEMS BIOLOGY IN THE STUDY OF NEUROLOGICAL DISORDERS: FOCUS ON ALZHEIMER’S DISEASE

Excessive alcohol use adversely affects brain functioning and is associated with certain neurological disorders. Although systems biology approaches are not yet being used extensively in the study of alcohol-related neurological disorders, they have been used in the study of other neurological conditions such as Alzheimer’s disease, which increasingly is becoming a major public health concern. Researchers using systems biological approaches to study the development and progression of this degenerative disease have discovered several genes whose expression already is altered in patients exhibiting the disease’s earliest stages. This article by Drs. Giulio M. Pasinetti and Susanne Hiller-Sturmhöfel discusses systems biology approaches, such as cDNA microarrays, protein array studies, and proteomic approaches, for studying Alzheimer’s disease, potential genes involved in its development, as well as the mechanisms underlying alcohol’s harmful effects on the brain and its consequences. (pp. 60–65)

## ALCOHOLIC LUNG DISEASE

Alcohol abuse has long been linked to an increased risk for lung infection (i.e., pneumonia). However, recent studies have shown that alcohol abuse also increases the risk of acute lung injury following major trauma, such as a serious motor vehicle accident, gunshot wound, or other event requiring hospitalization or when there is a risk of the spread of bacteria attributed to infection. This article by Drs. Corey D. Kershaw and David M. Guidot reviews recent studies that have found genetic analysis to be helpful in identifying potential candidate genes involved in alcohol-induced lung dysfunction, which might explain the newly identified association between alcohol abuse and acute lung injury in humans. (pp. 66–75)

## A SYSTEMS-BASED COMPUTATIONAL MODEL OF ALCOHOL’S TOXIC EFFECTS ON BRAIN DEVELOPMENT

Exposure of the growing fetus to alcohol in utero can cause a broad range of potential neurological disorders, ranging from alcohol-related neurodevelopmental disorder at the lowest exposure levels to fetal alcohol syndrome at the highest level. This article by Drs. Julia M. Gohlke, Susanne Hiller-Sturmhöfel, and Elaine M. Faustman discusses the effects of alcohol on nerve cell development (i.e., neurogenesis) and the formation of their connections with neighboring cells (i.e., synaptogenesis). Systems-based approaches, such as the generation of computational models to determine the relative effects of alcohol on various processes during brain development, can help to translate research findings obtained at a molecular or cellular level into assessment of risk associated with prenatal alcohol exposure. (pp. 76–83)

